# The impact of nocturnal road traffic noise, bedroom window orientation, and work-related stress on subjective sleep quality: results of a cross-sectional study among working women

**DOI:** 10.1007/s00420-021-01696-w

**Published:** 2021-05-27

**Authors:** Susanne Bartels, Mikael Ögren, Jeong-Lim Kim, Sofie Fredriksson, Kerstin Persson Waye

**Affiliations:** 1 Sleep and Human Factors Research, Institute of Aerospace Medicine, German Aerospace Center (DLR), Cologne, Germany; 2 School of Public Health and Community Medicine, Occupational and Environmental Medicine, Institute of Medicine, University of Gothenburg, Gothenburg, Sweden

**Keywords:** Poor sleep, Road traffic noise, Work stress, Additive interaction, Quiet façade

## Abstract

**Objective:**

To examine the effect of work-related stress and road noise exposure on self-rated sleep and potential additive interaction effects.

**Methods:**

Sleep and predictor variables were surveyed within two subsamples with 2191 and 1764 working women in a cross-sectional study. Sleep was assessed using a single question on general sleep quality and four questions on specific sleep problems and subsequently dichotomized (poor sleep vs. no poor sleep). Work-related stress was operationalized by *job strain* and *effort-reward imbalance*. Nocturnal exposure to road traffic noise was assessed as (a) the orientation of the bedroom window to a quiet façade vs. a low-, medium- or high-trafficked street and (b) energy-equivalent sound pressure levels for night-time modelled at the most exposed façade (*L*_night_). We distinguished between low (< 45 dB(A)), medium (45–50 dB(A)) and high exposure (> 50 dB(A)).

**Results:**

Poor sleep was associated with job strain and effort-reward imbalance. The prevalence of poor sleep did not increase with increasing *L*_night_, but bedroom window orientation showed a non-significant trend. A quiet façade had a protective effect on sleep in each *L*_night_ category. We found a non-significant trend for an additive interaction between bedroom window orientation and job strain.

**Conclusion:**

Noise levels modelled for the most exposed façade likely overestimate the actual exposure and thus may not be a precise predictor of poor sleep. Bedroom window orientation seems more relevant. Potential additive interaction effects between bedroom window orientation and job strain should be considered when interpreting epidemiological study results on noise-induced sleep disturbances.

**Supplementary Information:**

The online version contains supplementary material available at 10.1007/s00420-021-01696-w.

## Introduction

Restorative and undisturbed sleep is essential for physical and psychological well-being. Chronic sleep restriction and sleep disturbances have adverse effects on mood, cognitive functioning, and endocrine, metabolic, and cardiovascular systems (Banks and Dinges 2007; Basner et al. 2014; Colten, Altevogt, and Committee on Sleep Medicine and Research 2006a), and can ultimately result in increased risks for cardiovascular diseases, diabetes, obesity and psychological disorders, such as anxiety, depression, and alcohol use (Banks and Dinges 2007; Basner et al. 2014; Colten, Altevogt, and Committee on Sleep Medicine and Research 2006a). One reason for disturbed sleep, especially in urban environments, is transportation noise (Frei, Mohler, and Röösli 2014). According to the World Health Organization (WHO 2011), noise-induced sleep disturbances comprise the highest disease burden due to environmental noise exposure and road traffic noise is the most common source of transportation noise in the world. Exposure to nocturnal road traffic noise has been shown to cause acute sleep disturbance, using objective measures in both field and laboratory studies (Basner, Mueller, and Elmenhorst 2011; Sanok et al. 2018; Griefahn, Marks, and Robens 2006). An adverse effect of nocturnal road noise exposure has also been found in epidemiological studies and cross-sectional surveys where sleep disturbance is usually self-reported and noise exposure is most often modelled as energy-equivalent sound pressure levels for night-time (*L*_night_) at the most exposed façade (Basner and McGuire 2018).

During the last decade, the concept of a quiet façade has received more attention in studies. Previous publications have proposed that access to a quiet façade is important for road-noise-induced annoyance (Bodin, Bjork, Ardö, and Albin 2015; De Kluizenaar et al. 2013, 2011; Öhrstrom, Skanberg, Svensson, and Gidlof-Gunnarsson 2006; Van Renterghem and Botteldooren 2012). Similarly, there is evidence for a beneficial effect of a quiet façade for self-reported sleep quality (Bodin, Bjork, Ardö, and Albin 2015) and sleep disturbances attributed to road noise (Van Renterghem and Botteldooren 2012).

When investigating the association between exposure to road traffic noise and sleep, epidemiological survey studies often include demographical factors, such as sex, age and income (Brink 2011; Brown, Lam, and van Kamp 2015; Frei, Mohler, and Röösli 2014; Halonen et al. 2012; Bodin, Bjork, Ardö, and Albin 2015) and life-style factors, such as smoking, alcohol consumption, physical activity (Frei, Mohler, and Röösli 2014; Halonen et al. 2012; Bodin, Bjork, Ardö, and Albin 2015). However, the role of stress, in particular work stress, as an important predictor of poor sleep has mostly been neglected.

It is well established that psychological stress affects sleep (Akerstedt 2006). Work-related psychosocial stress has significantly increased over the last decades as a consequence of far-reaching changes in working life (Nuebling et al. 2013). A strong link between self-rated work stress and subjective sleep quality has emerged both in cross-sectional (Linton et al. 2015) and longitudinal studies (Akerstedt 2006; Linton 2004; Jansson and Linton 2006; Jansson-Fröjmark, Lundqvist, Lundqvist, and Linton 2007). According to a systematic review, job strain and imbalance between job effort and reward are important psychosocial predictors for decreased sleep 2011quality, poor sleep, and insomnia (Linton et al. 2015).

To the best of our knowledge, only one study, by Kristiansen et al. (2011), has investigated the effects of work stress and noise exposure on self-reported sleep quality in a multivariate model. The study found significant effects on sleep quality for job strain in both men and women and for road noise exposure in women. However, the study found no evidence of additive synergetic effects between job strain and noise exposure on sleep quality.

The present paper aims to expand the current knowledge by examining the effect of work stress (including job strain and effort-reward imbalance) and nocturnal road traffic noise exposure (including night-time equivalent levels and the orientation of the bedroom window) on self-rated poor sleep in multivariate models. Additionally, we examined potential synergetic effects of noise exposure and work-related stress. All analyses were conducted using data from an ongoing cohort of working women in Sweden (Fredriksson 2018; Fredriksson et al. 2019).

## Methods

### Sample

We analysed data from a questionnaire study including women with a preschool teachers’ degree issued between the years 1980 and 2012 from a university, and randomly selected women from the general population of the Västra Götaland County of Sweden born between 1943 and 1989. Questionnaires were sent out between October 2013 and July 2014 for parallel assessment of predictor and outcome variables in a cross-sectional study design. Response rates were 51% in the preschool teacher cohort and 38% in the general population (Fredriksson 2018).

Of a total of 11,167 valid responses (51% preschool teachers, 49% general population), 7575 women aged between 23 to 65 years were currently working part or full time. For 2191 of them, data on modelled nocturnal road noise levels and subjective sleep quality were accessible. This subsample is referred to as *subsample A* in the following. All respondents of subsample A were residing in the area of Gothenburg and its neighbouring community Mölndal.

**Fig. 1:**
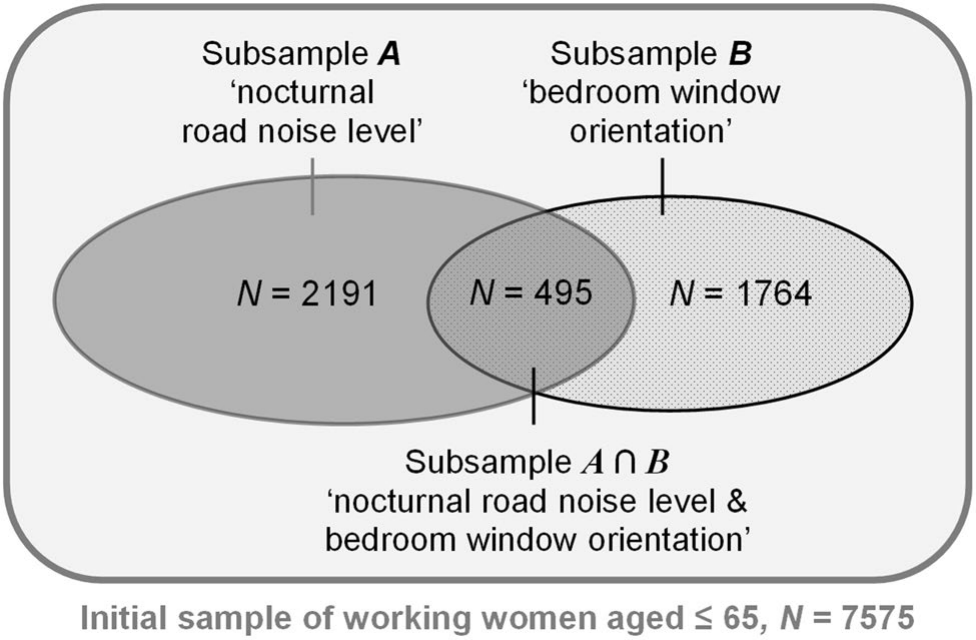
Outline of the subsamples investigated in the present study. The initial sample comprised *N* = 7575 working women aged ≤ 65 years

Due to an unfortunate mishap, the questionnaire sent out to the majority of the study population did not include the question on the orientation of the bedroom window. As a consequence, only 1764 respondents out of the whole sample of 7575 working females reported the orientation of their bedroom window and their sleep quality. This subsample, referred to as *subsample B*, included respondents from the area of Gothenburg and Mölndal and the whole Västra Götaland County. Information on both nocturnal road noise level and bedroom window orientation was obtainable for only 495 respondents from Gothenburg and Mölndal (subsample A ∩ B). They were included in both subsample A and subsample B. Fig. 1 outlines the subsamples investigated in this paper.

### Outcome measures

Sleep was measured using a single general question on self-rated sleep quality and a matrix of four questions on specific sleep problems in line with recommendations by Croy, Smith, Gidlöf-Gunnarsson, and Persson Waye (2017). General sleep quality was assessed by the single question “How would you rate your sleep?” with the response scale “very good”, “rather good”, “neither good nor bad”, “rather bad”, and “very bad”. The four questions about specific sleep problems were expressed as “During the past 12 months, how often … (i) have you had problems falling asleep in the evening, (ii) have you felt sleepy during the day, (iii) have you woken up too early and were unable to fall asleep again, and (iv) have you been tired in the morning?” Response options were “never/seldom”, “a few times per month”, “once per week”, “several times per week”, and “every day”. A factor analysis revealed all five questions loaded on a single factor (all factor loadings ≥ 0.68) with a satisfying internal consistency (Cronbachs’s α = 0.79). The five questions were therefore condensed and dichotomized as follows: When three or more of the five sleep questions had been answered with response options characterizing worse sleep (“rather bad” or “very bad” and “several times per week” or “every day”), sleep quality was coded to value 1 representing poor sleep. Otherwise, sleep was coded to 0 representing no poor sleep. The dichotomization of sleep quality using these cut-offs focuses on poor sleep at a degree of severity that affects the general perception of one’s sleep and day-time condition. To rule out that effects of individual sleep disturbances hide within the dichotomized categories, we additionally examined the effect of nocturnal road noise exposure and work-related stress on (a) problems falling asleep and (b) waking up too early and having problems falling asleep again in separate analyses as suggested by Evandt et al. (2017). These results were similar to those for the dichotomized poor vs. non-poor sleep variable. They are provided in supplementary materials.

### Modelled road noise exposure and orientation of the bedroom window

The nocturnal road noise levels were modelled according to the Nordic prediction method for road traffic noise (Jonasson and Nielsen 1996) based on geo coding of the respondent’s home address. The database for the noise calculations included geometries of roads, buildings, elevation data, ground types and noise barriers as well as traffic data on the number of standard and heavy vehicles and their distribution during night-time. We used the outdoor energy-equivalent night level (*L*_night_) for the time between 22:00 and 06:00, the standard in Sweden. Modelled noise data were calculated for the most exposed façade. No information was available regarding the floor in the building where the respondents lived. The effect of nocturnal road noise exposure on sleep was analysed as a continuous variable in a first step and as an ordinal variable in a second step. We differentiated between *L*_night_ below and above 45 dB(A) as the current night noise guidelines of the World Health Organization (WHO 2018) report potentially adverse effects on sleep at levels above *L*_night_ of 45 dB(A). In addition, we introduced a third category (> 50 dB(A)) indicating high exposure. Hence, the resulting categories were < 45 dB(A), 45–50 dB(A), and > 50 dB(A). Noise exposure data were only available for subsample A living in the Gothenburg and Mölndal area (*N* = 2191).

Information about the orientation of the bedroom window and, thus, about whether the bedroom window was situated on a quiet facade”, was obtained by the question “Does your bedroom have windows directly facing a street or road?” Answer options were “no street/road”, “yes, a low-traffic street/road”, “yes, a medium-traffic street/road”, and “yes, a high-traffic street/road”. Answer option “no street/road” was considered the equivalent of a quiet façade. Information on the bedroom window orientation was available for *N* = 1764 respondents living in the area of Västra Gotaland.

### Work-related stress

Work-related stress was operationalized by two separate constructs: (a) *job strain* and (b) *effort-reward imbalance*.

#### Job strain

**Fig. 2:**
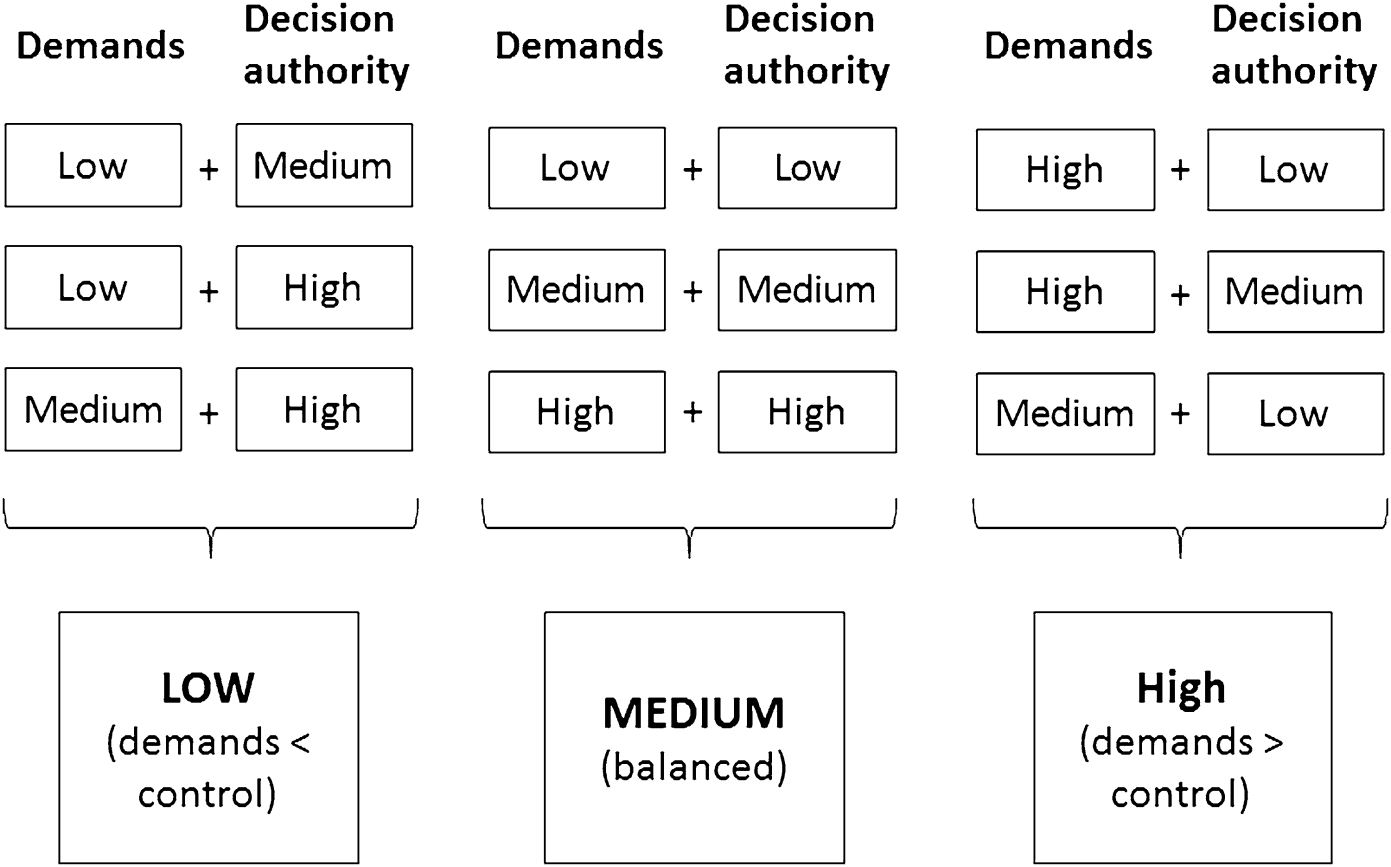
Construction of three categories of job strain (low, medium, high) based on the combination of different levels of demands and decision authority. Figure modified from Kristiansen et al. (2011)

The concept of *job strain* (Karasek 1979) describes mental job strain as a result of the interaction between psychological job demands and job control. *Job demands* refer to the workload and task requirements and *job control* refers to the ability of an individual to control his or her work activities with regard to the range of skills used on the job (skill discretion) and the individual’s authority to make work-related decisions (decision authority) (Van der Doef and Maes 1998). The Swedish Demand–Control–Support Questionnaire (DCSQ, Sanne, Torp, Mykletun, and Dahl 2005) was used. For the current study, we decided to not include skill discretion items in line with Kristiansen et al. (2011). Job demands were measured by five items and decision authority by two items. The response options for both subscales were “yes, often”, “yes, sometimes”, “no, seldom”, and “no, almost never”. We computed mean scores from five items describing job demands for all respondents with no more than one missing value. Missing values were imputed based on the mean score of the remaining job demands items. A mean score for decision authority was computed only when no item was missing. Assigning respondents to categories of low, medium and high job strain was based on a method described by Kristiansen et al. (2011), where different levels of demands and decision authority made up each category (Fig. 2). The levels were based on the mean scores as follows: 1–1.99 = low, 2–2.99 = medium, 3–4 = high.

#### Effort-reward imbalance

**Fig. 3:**
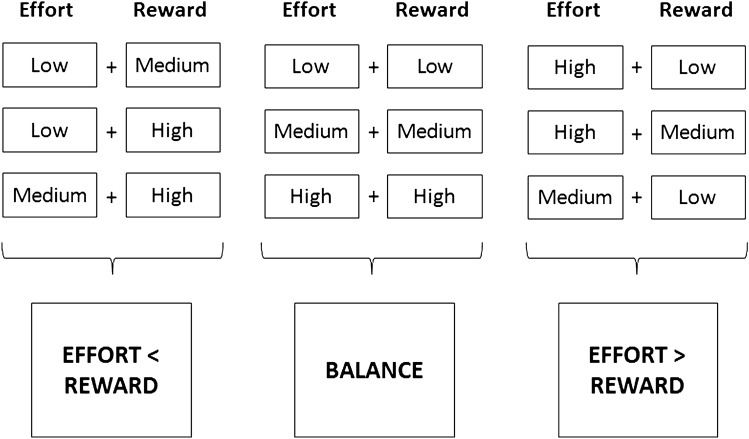
Construction of three categories of effort-reward (im-)balance (effort < reward, balance, effort > reward)

The concept of *effort-reward imbalance* (ERI, Siegrist 1996) focuses on the reciprocity of exchange between work-related effort (e.g., work pressure) and reward (e.g. promotion prospects, job security or insecurity). Imbalance is given under the condition “high cost/low gain” (Siegrist et al. 2004). Work-related effort and reward was measured by the Swedish short version of the Effort-Reward-Imbalance Questionnaire (Leineweber et al. 2010). We computed the effort-reward ratio using the following formula: ERI ratio = e/(r*c) where ‘e’ is the sum score of the effort scale, ‘r’ is the sum score of the reward scale and ‘c’ defines a correction factor depending on the number of items used in the questionnaire (Siegrist et al. 2004). In the current study, c was 3/7 as we used three items to measure effort and 7 items to measure reward. Sum scores were calculated only for respondents with no more than one missing value per scale. Missing values were imputed by scale mean scores. For consistency with the categorization of job strain, the relation between effort and reward was assessed in the same way as the relation between job demands and decision authority. This categorization was again based on mean scores. Scores between 1 and 1.99 defined “low”, 2–2.99 “medium”, and 3–4 “high” effort and reward, respectively. We categorized the ratio between effort and reward as follows in Fig. 3.

In line with prior research on ERI (e.g., Siegrist et al. 2004), we also analysed the effect of ERI on sleep using a dichotomous ERI variable with ratios between effort and reward ≤ 1.0 defined as “no imbalance” and ratios > 1.0 as “imbalance”. The results using this dichotomous ERI variable were similar to the results for the categorical ERI variable. We therefore only report the latter.

### Confounders and modifiers

Several demographic and lifestyle factors have a priori been identified as potential modifiers or confounders of the relationship among work stress, nocturnal exposure to road noise and poor sleep (Table 1). We controlled for current smoking, alcohol consumption, obesity via BMI, and age, since these are risk factors for sleep disturbance, such as obstructive sleep apnea syndrome (OSAS), and can deteriorate sleep quality and sleep architecture (Wetter, Young, Bidwell, Badr, and Palta 1994; Young et al. 1993; Bresnitz, Goldberg, and Kosinski 1994; Colten, Altevogt, and Committee on Sleep Medicine and Research 2006b). Physical activity was controlled for due to the beneficial effects of regular exercises on sleep quality (Kredlow, Capozzoli, Hearon, Calkins, and Otto 2015). These variables were considered potential modifiers of the hypothesized relationship between road noise exposure and poor sleep. We additionally included self-rated noise sensitivity assessed via a four-point verbal answer scale ranging from “not at all” to “very” and regarded as interval scale, as it was previously shown to modify the effect of noise exposure on self-rated sleep disturbance (Miedema and Vos 2003; Basner and McGuire 2018). Noise sensitivity may also have acted as a confounder since it is not only affecting sleep disturbance ratings but also influencing the choice of less noisy living areas. The same was expected for educational level and monthly income which were treated as confounders (Kristiansen et al. 2011; Frei, Mohler, and Röösli 2014; Evandt et al. 2017). To account for potential biases due to sampling participants from two separate cohorts, i.e. the preschool teacher cohort and the general population cohort, a binary variable was included as a possible confounder.

**Table 1:** Distribution of demographic and lifestyle factors within the sample

Sample characteristics	Distribution in subsample A (*N* = 2191)	Distribution in subsample B (*N* = 1764)
*Age* (mean ± SD)	46.0 ± 10.6	47.2 ± 10.0
*Educational level* (number, %)		
Lower secondary or lower	76 (3.5)	92 (5.2)
Upper secondary	299 (13.7)	351 (20.0)
Higher than upper secondary	1814 (82.9)	1311 (74.7)
*Monthly household income in SEK* (number, %)		
< 30,000	531 (24.0)	362 (20.8)
≥ 30,000	1650 (76.0)	1377 (79.2)
*Cohort* (number, %)		
Pre-school teacher	1067 (48.7)	842 (47.7)
Other occupation	1124 (51.3)	922 (52.3)
*Noise sensitivity (mean* ± *SD)*	2.3 ± 0.7	2.2 ± 0.7
*BMI* in kg/m^2^ (number, %)		
Normal (18.5–24.99)	1365 (63.1)	1035 (59.6)
Underweight (< 18.5)	30 (1.4)	18 (1.0)
Overweight (25–34.99)	722 (33.4)	641 (36.9)
Obese (≥ 35)	47 (2.2)	42 (2.4)
*Physical activity* (number, %)		
Easier or more exhaustive exercises at least a couple of hours a week	1830 (84.1)	1505 (85.8)
Mostly sedentary	290 (13.3)	208 (11.9)
Hard training several times a week	56 (2.6)	41 (2.3)
*Current smoking (number, %)*		
No, never / no, formerly	2035 (93.1)	1631 (93.1)
Yes	151 (6.9)	120 (6.9)
*Alcohol intake* (number, %)		
Never / once per months or more rarely	708 (32.4)	643 (36.7)
2–4 times per months	980 (44.9)	824 (47.1)
2–3 times per week	459 (21.0)	265 (15.1)
4 times a week or more often	35 (1.6)	19 (1.1)

### Statistical analysis

Mean values with standard deviation (SD) were calculated for continuous variables and frequencies expressed as percentages were used for categorical variables. All statistical calculations were conducted using IBM SPSS Statistics for Windows, version 25 (IBM Corp., Armonk, N.Y., USA). Separate models were computed for the impact of nocturnal road noise exposure and bedroom window orientation on poor sleep given that the data came from two separate subsamples (A and B). We calculated univariate logistic regression analyses between the main predictors (either nocturnal exposure to noise or work stress) and the outcome sleep (poor sleep vs. no poor sleep), see column *Univariate model* in Table 2 and 3. Effects estimates were presented as Odds Ratios (OR) with 95% confidence intervals (CI). Statistical significance was assumed when the 95% confidence intervals for the Odds Ratios did not include 1 or for a *p*-value < 0.05 with *p* values < 0.1 indicating a non-significant trend.

**Table 2:** Effect of night-time road noise exposure level and work stress on sleep. Univariate and adjusted Odds Ratios (*OR*) with 95% Confidence Intervals (*CI*), subsample A

			Univariate model		Adjusted model I (*job strain*)		Adjusted model II (*ERI*)	
Variable and level	*n* with poor sleep	*n* without poor sleep	OR	95% CI	OR	95% CI	OR	95% CI
*Nocturnal road noise*								
Low (< 45 dB, reference)	139	570	1.00		1.00		1.00	
Medium (45–50 dB)	120	544	0.90	0.69–1.19	0.96	0.73–1.28	0.96	0.72–1.28
High (> 50 dB)	112	607	0.76*	0.58–0.99	0.80	0.60–1.07	0.81	0.61–1.08
*Job strain*								
Medium/balanced (reference)	179	765	1.00		1.00			
Low	94	730	0.55***	0.42–0.72	0.62***	0.47–0.82		
High	98	226	1.85***	1.39–2.47	1.83***	1.35–2.47		
*ERI*								
Balance (reference)	88	551	1.00				1.00	
Effort < reward	12	263	0.29***	0.15–0.53			0.33***	0.17–0.61
Effort > reward	271	907	1.87***	1.44–2.43			1.71***	1.30–2.25

*p < .05; **p < .01; ***p < .001. Adjusted models I and II control for the following potential confounders and modifiers: age, educational level, monthly family income, type of the cohort, body mass index, BMI, physical activity, current smoking, alcohol, and noise sensitivity

**Table 3:** The effect of bedroom window orientation and work stress on sleep. Univariate and adjusted Odds Ratios (*OR*) with 95% Confidence Intervals (*CI*), subsample B

			Univariate model		Adjusted model I (*job strain*)		Adjusted model II (*ERI*)		
Variable and level	*n* with poor sleep	*n* without poor sleep	*OR*	95% CI	*OR*	95% CI	*OR*		95% CI
*Bedroom window orientation*									
No street (reference)	165	919	1.00		1.00		1.00		
Street with low traffic	65	375	0.97	0.71–1.32	0.91	0.65–1.26	0.97		0.70–1.34
Street with medium or high traffic	31	106	1.63*	1.06–2.51	1.40	0.89–2.22	1.53^+^		0.97–2.42
*Job strain*									
Medium/balanced (reference)	134	521	1.00		1.00				
Low	72	717	0.39***	0.29–0.53	0.45***	0.33–0.62			
High	55	162	1.32	0.92–1.89	1.38^+^	0.94–2.02			
*ERI*									
Balance (reference)	53	476	1.00				1.00		
Effort < reward	24	274	0.79	0.47–1.30			0.87		0.52–1.46
Effort > reward	184	650	2.54***	1.83–3.53			2.39***		1.70–3.35

^+^*p* < .1, *p < .05, **p < .01, ***p<.001. Adjusted models I and II control for the following potential confounders and modifiers: age, educational level, monthly family income, type of the cohor t, body mass index, BMI, physical activity, current smoking, alcohol, and noise sensitivity

For multivariate logistic regression models, one work stress variable (either job strain or ERI) and one noise exposure variable (either bedroom window orientation or *L*_night_) were included, controlling for a priori selected confounders/modifiers (i.e. all variables described in Sect. Confounders and modifiers). Job strain and ERI were analysed in separate models, due to their high intercorrelation (Spearman’s *r* > 0.43, *p* < 0.001). Accordingly, a total of four adjusted models were computed, see columns *Adjusted model I* and *II* in Table 2 and 3, each.

### Testing for synergetic effects

We assessed synergetic interaction effects between work stress and exposure to nocturnal road noise on an additive scale. Positive departure from additivity of effects implies that the number of cases (respondents reporting poor sleep) attributable to high levels of two risk factors (nocturnal noise exposure and work stress) in combination is larger than the sum of the numbers of cases that would be caused by high levels of each risk factor (Richardson and Kaufman 2009, p. 756). The amount of interaction was quantified by attributable proportion, AP (Hosmer and Lemeshow 1992). In absence of interaction, AP is 0. AP > 0 indicates an additive interaction between a high expression of the two risk factors. In accordance with the paper by Kristiansen et al. (2011), we calculated AP’s as recommended by Hosmer and Lemeshow (1992) using OR instead of RR. For the estimation of their confidence intervals, we used a spreadsheet proposed by Knol and VanderWeele (2012) which is based on the Hosmer–Lemeshow procedure (1992). This calculation of AP’s applies to dichotomous variables. Therefore, the calculations of APs involving variables with three categories were carried out for the highest and lowest levels of these variables.

Synergetic interaction effects between noise exposure and work stress variables were examined when these two variables showed at least a trend for an increasing effect on poor sleep.

## Results

### Demographic and lifestyle characteristics of the study population

Characteristics of the study population are shown in Table 1. Distribution of demographic and lifestyle characteristics did not differ between subsample A and subsample B.

### Poor sleep and nocturnal road noise exposure

**Fig. 4:**
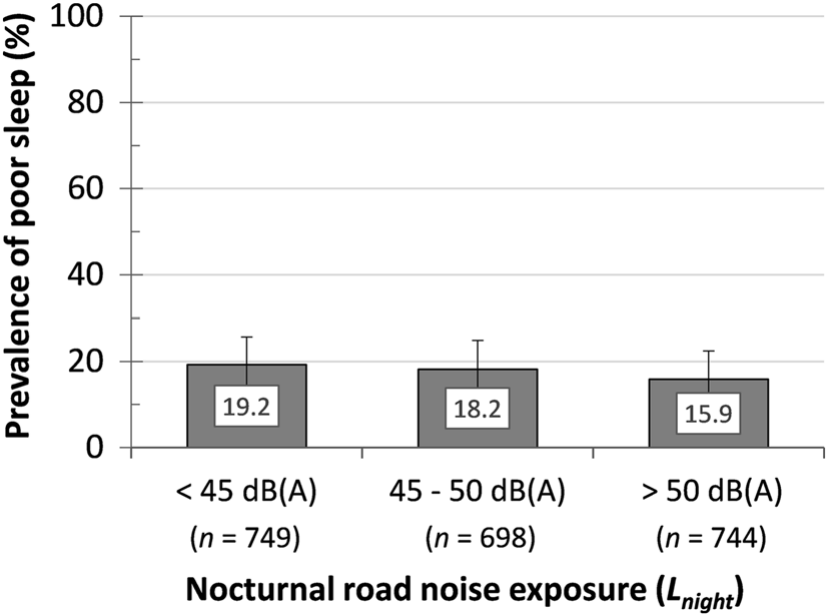
Distribution of *poor sleep* in three categories of the nocturnal road noise exposure (*L*_night_), *N* = 2191 (subsample A) based on descriptive univariate analyses. In parentheses: Number of respondents per exposure category. *L*_night_ data were accessible only for respondents with residence in Gothenburg and Mölndal

Figure 4 shows the distribution of self-reported poor sleep among three categories of nocturnal road noise exposure operationalized as *L*_night_ (based on descriptive univariate analyses). Contrary to the expected positive relationship, we observed a negative association between *L*_night_ and prevalence of poor sleep.

**Fig. 5:**
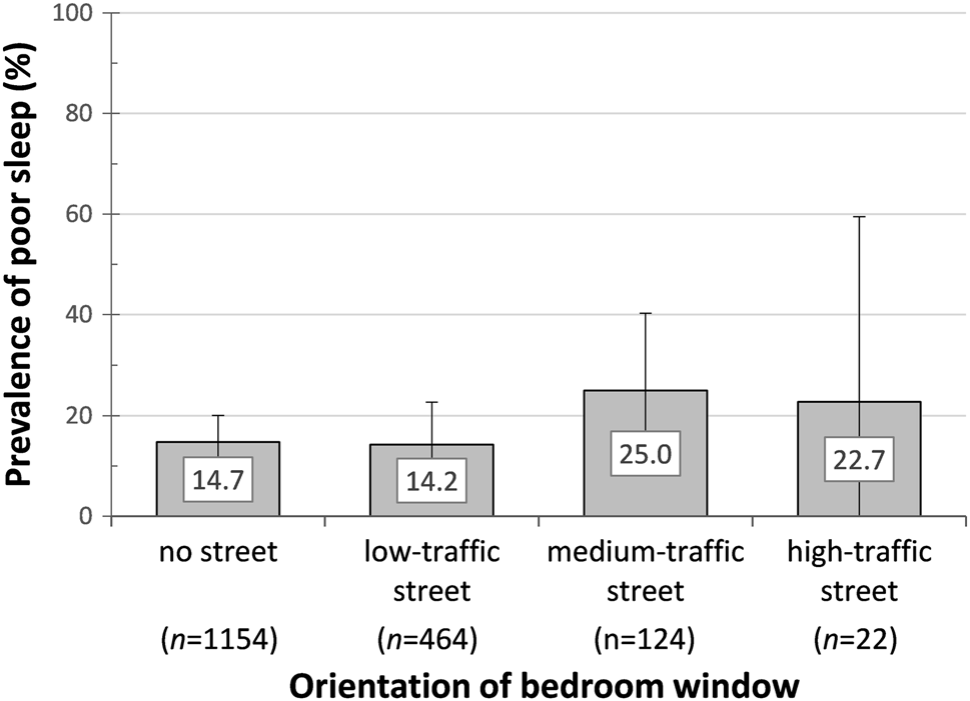
Distribution of *poor sleep* in four categories of bedroom window orientation, *N* = 1764 (subsample B) based on descriptive univariate analyses. In parentheses: Number of respondents per exposure class

Figure 5 shows the prevalence of poor sleep in the four categories of bedroom window orientation *no street*, *low-traffic street*, *medium-traffic street*, and *high-traffic street* (based on descriptive univariate analyses). The percentage of respondents indicating poor sleep increased particularly among *medium-traffic street* (25.0%) and *high-traffic street* (22.7%). As shown in Fig. 5, the sample size in the four categories differed greatly. For this reason and as the prevalence of poor sleep differed only marginally in these two categories, *medium* and *high* were combined for all following analyses.

**Fig. 6:**
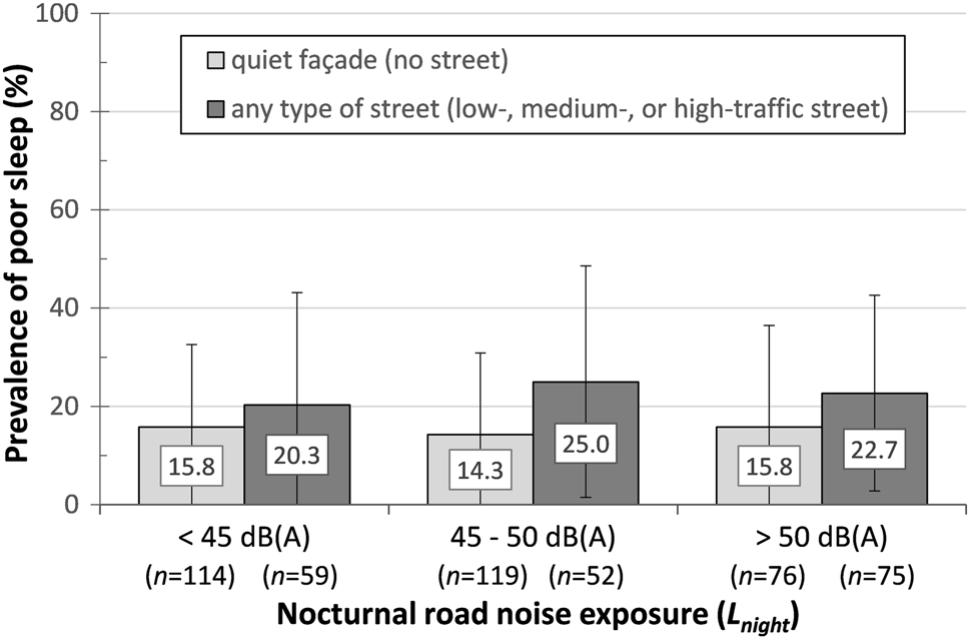
Distribution of *poor sleep* in three categories of the nocturnal road noise exposure (*L*_*night*_) in consideration of the bedroom window orientation, *N* = 495 (subsample A ∩ B) based on descriptive analyses without considering modifying or confounding variables. In parentheses: Number of respondents per category. *L*_night_ data were accessible only for respondents with residence in Gothenburg and Mölndal

A benefit of having a quiet-facade bedroom window was observed in a subsample with *N* = 495 respondents (subsample A ∩ B) for whom both data on bedroom window orientation and *L*_night_ were available. Figure 6 shows that the prevalence of poor sleep was lower among respondents with a quiet bedroom façade at all three *L*_night_ categories. The benefit was largest (10.3%) in residents exposed to an *L*_night_ between 45 and 50 dB(A).

### Association among exposure to road noise, work-related stress, and poor sleep

There was a trend towards a negative association between the *L*_night_ and poor sleep. A crude model with the *L*_night_ as a continuous variable showed a non-significant trend for an effect on poor sleep (OR per 1 dB-step = 0.99, CI 0.98–1.00). Results remained unaltered when (i) analysing *L*_night_ as an ordinal variable, (ii) including job strain, confounders, and modifiers; and (iii) including ERI, confounders, and modifiers (Table 2). Contrary to our hypotheses, respondents in the *high L*_night_ category reported slightly lower rates (15.9%) of poor sleep than respondents in the low *L*_night_ category (19.2%) as shown in Fig. 4.

When the *L*_night_ was categorized according to the suggestion of the World Health Organization (WHO 2018) differentiating between *L*_night_ < 45 dB and ≥ 45 dB, we found a non-significant difference between the two cateogries in a univariate model. The prevalence of poor sleep was slightly higher in the exposure group with *L*_night_ < 45 dB (19.2%) than in the exposure group with *L*_night_ ≥ 45 dB (17.0%, OR = 0.860, CI 0.685–1.080). The effect remained non-significant both when including job strain, confounders, and modifiers (OR = 0.883, CI 0.692–1.128) and when including ERI, confounders, and modifiers (OR = 0.895, CI 0.701–1.142) in multivariate models.

Table 3 shows a positive association between poor sleep and the bedroom window orientation. Both the univariate model including bedroom window orientation as the only predictor and the adjusted model including ERI and job strain revealed an increase in the prevalence of poor sleep between the no street and the medium/high-traffic street condition; effects were, however, not consistently significant across models.

As evidenced in Tables 2 and 3, job strain was associated with self-rated poor sleep. Low job strain reduced the prevalence of poor sleep compared to medium job strain in all univariate and adjusted models. High job strain increased the prevalence of poor sleep compared to medium job strain on a significant level in the models for subsample A, but not significantly in the models for subsample B.

We also observed an association between the effort-reward ratio and poor sleep (see Tables 2 and 3). Effort-reward imbalance significantly increased the prevalence of poor sleep compared to effort-reward balance in all univariate and adjusted models. The prevalence of poor sleep was significantly reduced when self-rated effort was smaller than reward in the models of subsample A, but not in the models of subsample B.

### Synergetic effects of exposure to road noise and work-related stress

Exceedance from additivity was examined between the predictor’s *bedroom window orientation* and *job strain* and *ERI* in three categories, respectively. The *L*_night_ was not included in these analyses since it did not show any trend with a positive association to the prevalence of poor sleep. As shown in Table 4, we found a non-significant interaction exceeding additivity among respondents with high job strain in combination with a bedroom window facing a medium or high-traffic street. The Attributional Proportion (AP) was positive and suggested a non-significant trend (AP = 0.46, CI  –0.09 to 1.00). The combination between the imbalance condition effort > reward and a bedroom window facing medium or high-traffic street, also indicated positive departure from additivity, albeit not on a statistically significant level (AP = 0.12, CI  – 0.52 to 0.76).

**Table 4:** Analysis of additive interaction effects between bedroom window orientation and work stress. The distribution of levels of bedroom window orientation and work stress in respondents with vs. without poor sleep refer to crude numbers (n)

	No street (quiet façade)				Medium-/high-traffic street				Attributional Proportion	
	*n* with poor sleep	*n* without poor sleep	*OR*	95% CI	*n* with poor sleep	*n* without poor sleep	*OR*	95% CI	*AP*	95% CI
Low job strain	47	474	1.00		6	52	1.09	0.43–2.75	0.46	− 0.09–1.00
High job strain	31	98	3.02	1.79–5.09	9	14	5.71	2.24–14.56		

Effort < reward	14	159	1.00		3	23	1.33	0.35–5.12	0.12	− 0.52–0.76
Effort > reward	117	446	2.19	1.19–4.01	19	49	2.86	1.28–6.43		

Estimates for odds ratios (OR) and confidence intervals (CI) are adjusted for the effect of a priori selected confounders and modifiers (Sect. Confounders and modifiers)

## Discussion

Exposure to both nocturnal road noise and work-related strain had an impact on the prevalence of self-rated poor sleep in women; yet, compared to work-related stress, the impact of noise exposure was marginal. Exposure variables bedroom window orientation and the *L*_night_ were not consistently positively associated with the prevalence of poor sleep. Albeit non-significant, a trend for synergetic interaction effects was observed between nocturnal road noise exposure and work-related stress.

### Strengths and limitations

We assessed poor sleep using questions on general sleep quality as well as on specific symptoms of disturbed sleep (such as problems to fall asleep) for a more global assessment of poor sleep (Croy, Smith, Gidlöf-Gunnarsson, and Persson Waye 2017). The items were neutral, i.e., not referring to road noise as the source for sleep disturbances. A recent meta-analysis (Basner and McGuire 2018) found an effect of the context of the question, i.e. whether road noise was explicitly named as a potential noise source. Compared to the number of studies investigating sleep disturbance attributed to road traffic noise, however, relatively few studies have been published on general sleep disturbance (Basner and McGuire 2018). Our assessment of general sleep disturbances reduced the likelihood of personal evaluations and biases due to attitudes towards the noise source. The cut-off we applied for poor sleep was relatively restrictive with ≥ 3 out of 5 questions answered with at least the second highest response option. However, a less restrictive cut-off criterion (≥ 2 out of 5 questions answered by the two highest response options) or a cut-off based on the mean score across all five sleep questions showed similar coefficients for noise exposure and work stress variables.

With regard to the categorization of work-related stress, we did not only compare two groups of job strain (i.e. high versus low) and ERI (imbalance versus no imbalance), but defined a third category allowing for a more granular evaluation of job strain and ERI levels. The categorization was conducted according to a priori defined cut-off values (i.e. sum scores). This procedure is preferable to a categorization based on quantile splits, which may vary between study populations and survey waves (Hadžibajramović 2015). Regarding the interpretation of the effects of work-related stress on poor sleep, some limitations must be noted. Data were collected in a cross-sectional study design and, thus, reverse causality cannot be ruled out.

Apart from noise exposure and work-related stress, other factors may exist that influenced sleep, notably sleep-disturbed breathing due to obstructive sleep apnea syndrome (OSAS). Although we did not explicitly screen for this syndrome, we adjusted our results for known risk factors of sleep-disturbed breathing, i.e. current smoking, alcohol consumption, obesity (via Body Mass Index), and age (Wetter, Young, Bidwell, Badr, and Palta 1994; Young et al. 1993; Bresnitz, Goldberg, and Kosinski 1994). The latter has a strong impact on sleep architecture (Colten, Altevogt, and Committee on Sleep Medicine and Research 2006b). Nevertheless, univariate models and models adjusting for confounders and modifiers did not differ remarkably regarding the effect on poor sleep due to road noise exposure and work-related stress.

We operationalized nocturnal road noise exposure based on the orientation of the bedroom window and the modelled *L*_night_ for outdoors at the most exposed façade. We originally intended to analyse the association between the *L*_night_ and sleep quality taking into account the bedroom window orientation, as suggested by Evandt et al. (2017). However, the majority of the sample received a questionnaire that did not include the question on the orientation of the bedroom window. Consequently, combined *L*_night_ and bedroom façade data were available for a subsample of *N* = 495 respondents. This sample was too small to provide the necessary statistical power for the intended analyses on the hypothesized synergetic effect of noise exposure and work-related stress on sleep. Therefore, the *L*_night_ of the most exposed façade served as proxy for the nocturnal exposure level without controlling for the actual bedroom façade. Assuming that residents living on noisy roads are likely to choose more shielded sides of their homes for their bedrooms (Frei, Mohler, and Röösli 2014), this major limitation might have led to an underestimation of the true effect of the equivalent continuous outdoor level on sleep. In fact, the vast majority of respondents who received the question on bedroom window orientation reported that their bedrooms faced no street or a low-traffic street. From the *N* = 495 respondents with information on both *L*_night_ and bedroom window orientation, 62% reported having a quiet bedroom façade. Thus, for those respondents with a window facing no street, the *L*_night_ may have been overestimated, and the observed association between road noise exposure might be biased.

Information where and on which floor in the building respondents lived was not assessed and hence not accounted for in the noise exposure estimation. Furthermore, we did not have information on window positions. Depending on the window position, median outdoor-indoor level differences lie between 10 dB(A) for open window and 28 dB(A) for closed windows (Locher et al. 2018). It has previously been reported that people keep their bedroom windows closed more often when nocturnal road noise levels are high (Öhrstrom, Skanberg, Svensson, and Gidlof-Gunnarsson 2006). We did not have information on maximum levels, which are an important factor for disturbed and fragmented sleep (Basner and McGuire 2018). Using a combination of self-reported bedroom window direction, window-opening behaviour and advanced modelled noise exposure, where façade insulation and maximum level are being taken into account, may help improve the accuracy of noise exposure modelling.

The answer “no street/road” for bedroom window orientation was used as a surrogate measure for a quiet façade, since we assumed that road traffic was the dominant noise source in our sample. However, noise sources other than road traffic may be present at supposedly quiet facades, such as railways, industrial fields, or clubs and bars, about which no data were available.

### Interpretation

We found a non-significant trend for an increase in the prevalence of self-reported poor sleep among respondents with a bedroom window facing a medium or high-traffic street compared to those with a bedroom window facing no street. No difference in the prevalence of poor sleep was found between the two conditions bedroom window facing no street (= quiet façade) and bedroom window facing a low traffic street. It seems plausible that being exposed to a self-rated low traffic street is associated with low-level night-time noise in the studied areas, thus causing no perceivable sleep impairments. Yet, facing no street does not automatically imply a quiet façade, such as a green space, yard or garden, or water, as described by Bodin, Bjork, Ardö, and Albin (2015). Importantly, despite these limitations, results showed a beneficial effect of a quiet bedroom façade on sleep quality, irrespective of the modelled noise level. This result is in line with prior findings (Bodin, Bjork, Ardö, and Albin 2015; De Kluizenaar et al. 2013; Öhrstrom, Skanberg, Svensson, and Gidlof-Gunnarsson 2006; Van Renterghem and Botteldooren 2012). With regard to absolute figures for noise exposure, Öhrstrom, Skanberg, Svensson, and Gidlof-Gunnarsson (2006) defined a quiet façade as an average night noise level < 45 dB(A). Interestingly, we found an effect of the bedroom window orientation (i.e. facing no street compared to any type of street) in respondents exposed to a modelled outdoor *L*_night_ < 45 dB(A). This finding suggests a benefit of having access to a quiet façade even in generally low exposed areas.

Increasing modelled *L*_night_ levels did not increase the prevalence of self-reported poor sleep. Instead we observed a trend for a negative association. The most plausible explanation for this finding seems to be the above-mentioned limitations in the modelling of the *L*_night_ at the most exposed façade. As discussed earlier, a major part of the respondents’ bedroom window was not directed to the most exposed façade, but rather a quiet façade. As a consequence, the actual nocturnal road noise level was most likely misclassified.

Moreover, equivalent continuous levels generally have limited explanatory power for sleep disturbances, especially when the noise is intermittent and not continuous (Griefahn et al. 2000; Wunderli et al. 2016). Different noise scenarios may result in the same equivalent level (Basner and McGuire 2018), such as few, but loud road noise events emerging from the background level vs. a rather continuous noise scenario with many soft events. However, the likelihood for nocturnal awakenings and sleep fragmentation may be higher in the first scenario as awakening probabilities strongly depend on maximum levels and their relation to the background noise (Sanok et al. 2018).

The wording of the questions on sleep quality and sleep disturbance may in part account for the (absent) association between *L*_night_ and self-rated poor sleep. Basner and McGuire (2018) reported that road noise night levels were significantly associated with self-rated high sleep disturbance when questions explicitly referred to road noise as affecting sleep. When sleep disturbances were assessed without reference to road noise as a potential source for disturbed sleep, only a non-significant, very small association between noise level and sleep disturbances was reported. In addition, a recent study showed that the strength of association between modelled road noise exposure and self-reported sleep quality differed between men and women. While a significant association was observed in men, none was found in women (Röösli, Mohler, Frei, and Vienneau 2014). Since only women were included in this survey, generalizability of the present findings to the whole population should be treated with caution.

In line with prior studies on the effect of work-related stress on sleep (Akerstedt 2006; Fahlén et al. 2006; Ota et al. 2009; Linton et al. 2015; Akerstedt, Nordin, Alfredsson, Westerholm, and Kecklund 2012; Kristiansen et al. 2011), we found a significant influence of both job strain and ERI on self-reported sleep quality. High job strain and an imbalance between effort and reward were associated with a higher prevalence of poor sleep, compared to medium job strain or a balanced effort-reward ratio, although not consistently significant across models. In addition, prevalence of poor sleep decreased when control exceeded job demands (= low job strain) and when reward exceeded effort, albeit not significant in all analyses. In work-related contexts, a balanced ratio between (high) demands and (high) control is assumed to elicit positive consequence, such as learning new behaviour on an active job (Karasek 1979). With regard to sleep, however, our findings suggest that a surplus of control is preferable. The same applies to the ratio between effort and reward. However, since data came from a cross-sectional study, causality cannot be established. Reverse causality between work-related strain and poor sleep is also conceivable and would suggest that persistently poor sleep and resulting performance decrements can affect the experience of the work environment (Linton et al. 2015).

In addition to independent effects for bedroom window orientation (i.e., on a trend level) and job strain, we found a non-significant trend for an additive interaction between these two factors. A bedroom window orientation towards a medium or high-traffic street in combination with a high level of job strain showed a more than additive risk for self-rated poor sleep. Non-significant positive departure from additivity was also found for the combination of a bedroom window orientation towards a medium or high-traffic street and ERI.

Of the three categories of noise exposure and work-related stress, we only included the extreme categories and left out the middle category for the analysis of additive interaction. This approach was chosen for an easier interpretation of results and stronger contrasts. Combining the middle category with the extreme categories would probably have blurred the effect size of interaction (Kristiansen et al. 2011). In summary, our findings suggest that (very) high or (very) low levels of subjective job strain may interact on an additive scale with road noise exposure assessed via the orientation of the bedroom window. Kristiansen et al. (2011), who first focussed on potential synergetic interactions between nocturnal road noise exposure and job strain, did not find positive departure from additivity among women. However, their findings are only partially comparable to our findings as Kristiansen et al. operationalized noise exposure by modelled *L*_night_ and not by bedroom window orientation.

In general, the demographic and lifestyle characteristics in subsample A (sample with *L*_night_ values available, from Gothenburg municipality and Mölndal) and subsample B (sample with information on bedroom window orientation from all over Västra Götaland County) differed only slightly and we do not expect a severe bias in subjective poor sleep from these differences.

## Conclusion

Work-related stress assessed as job strain and effort-reward imbalance and at least on a trend level also bedroom window orientation both affected self-rated poor sleep in working women. A non-significant trend for an additive interaction between work-related stress and bedroom window orientation on poor sleep was observed, suggesting that work-related stress may be an important factor in studies on the effect of transportation noise on sleep and should be taken into account in future research. In view of previously reported sex differences in the relation between road noise exposure and sleep that showed a relation in men but not in women (Röösli, Mohler, Frei, and Vienneau 2014), our findings in a female sample warrant more research to establish generalizability on a population level including male respondents.

The average outdoor night noise level modelled at the most exposed façade (*L*_night_) was a poor predictor of self-reported sleep quality most likely because it does not represent an appropriate proxy for the sleeper′s actually perceived noise exposure. The bedroom window orientation, specifically towards a quiet façade, seems to play a more important role. Sleeping in a quiet-façade bedroom had a protective effect on subjective sleep quality for all *L*_night_ categories and even in areas with generally low road noise exposure. Future studies on the effect of nocturnal road noise exposure should include the orientation of the bedroom window in their modelling of outdoor noise levels.

## Outlook

The findings of the present paper refer to cross-sectional analyses coming along with limitations regarding the causality of effect. However, they raise some questions on work-related stress and exposure classifications for improved knowledge on factors affecting poor sleep and health. These questions warrant following up preferably in longitudinal studies.

Although subjective sleep measures have often been found to be at least moderately correlated with objective sleep measurements, such as polysomnography (Croy, Smith, Gidlöf-Gunnarsson, and Persson Waye 2017; Griefahn et al. 2006), several limitations should be considered (Basner, Brink, and Elmenhorst 2012). Subjective measures do not allow for the detection of subtle and non-conscious physiological changes, such as sleep stage changes from deeper to lighter sleep. Objectively measured changes in sleep architecture as well as (non-conscious) fragmentation of sleep have been demonstrated for both work-related stress (see Akerstedt 2006) and transportation noise (see Basner and McGuire 2018). Hence, future studies are encouraged to investigate potential synergetic effects between these two factors on objectively assessed sleep.

Previous studies have reported a slightly increased risk for cardiovascular diseases associated with chronic nocturnal traffic noise exposure (Basner et al. 2014; van Kempen, Casas, Pershagen, and Foraster 2018). This association may be due to repeated vegetative and cortical arousals and resulting disturbances of sleep and metabolic regulations due to traffic noise events (Babisch 2011; Basner et al. 2014; Basner, Brink, and Elmenhorst 2012). Taken together with previously reported associations between high work-related stress and prevalence of cardiovascular diseases (Kivimäki et al. 2012; Siegrist 1996; Van der Doef and Maes 1998), additive interaction effects between transportation noise and work stress on the risk for cardiovascular diseases warrant further investigations.

## Supplementary Information

Below is the link to the electronic supplementary material.

Supplementary file1 (DOCX 34 KB)

## Data Availability

The datasets analysed during the current study are not publicly available.
