# Supplementary material for: The impact of nocturnal road traffic noise, bedroom window orientation, and work-related stress on subjective sleep quality: results of a cross-sectional study among working women
Source: Int Arch Occup Environ Health. 2021 May 27;94(7):1523–36. doi: 10.1007/s00420-021-01696-w (PMC8384796; doi:10.1007/s00420-021-01696-w)
Supplement: Supplementary file 1 — Supplementary file1 (DOCX 34 KB) [file 420_2021_1696_MOESM1_ESM.docx]

**Supplementary Table 1** Effect of nocturnal road noise exposure and work-related stress (Job strain or ERI) on problems falling asleep, within subsample A, showing univariate and adjusted Odds Ratios (*OR*) with 95 % Confidence Intervals (*CI*).

|  | Problems falling asleep | | Univariate model | | |  | Adjusted model I  (*job strain*) | | |  | Adjusted model II  (*ERI*) | | |
| --- | --- | --- | --- | --- | --- | --- | --- | --- | --- | --- | --- | --- | --- |
| Variable and level | *n* with  problems | *n* without problems | OR | | 95 % CI |  | OR | | 95 % CI |  | OR | | 95 % CI |
| *Nocturnal road noise* |  |  |  |  |  |  |  |  |  |  |  |  |  |
| Low (< 45 dB, reference) | 90 | 617 | 1.00 |  |  |  | 1.00 |  |  |  | 1.00 |  |  |
| Medium (45 - 50 dB) | 90 | 573 | 1.08 |  | 0.79-1.47 |  | 1.11 |  | 0.80-1.53 |  | 1.11 |  | 0.80-1.53 |
| High (> 50 dB) | 84 | 635 | 0.91 |  | 0.66-1.25 |  | 0.94 |  | 0.68-1.31 |  | 0.96 |  | 0.69-1.33 |
|  |  |  |  |  |  |  |  |  |  |  |  |  |  |
| *Job strain* |  |  |  |  |  |  |  |  |  |  |  |  |  |
| Medium/balanced (reference) | 128 | 815 | 1.00 |  |  |  | 1.00 |  |  |  | . |  |  |
| Low | 67 | 757 | 0.56 | ^***^ | 0.41-0.77 |  | 0.57 | ^***^ | 0.41-0.78 |  | . |  | . |
| High | 69 | 253 | 1.74 | ^***^ | 1.25-2.40 |  | 1.55 | ^*^ | 1.10-2.17 |  | . |  | . |
|  |  |  |  |  |  |  |  |  |  |  |  |  |  |
| *ERI* |  |  |  |  |  |  |  |  |  |  |  |  |  |
| Balance (reference) | 56 | 582 | 1.00 |  |  |  | . |  |  |  | 1.00 |  |  |
| Effort < reward | 15 | 260 | 0.60 | ^+^ | 0.33-1.08 |  | . |  | . |  | 0.65 |  | 0.36-1.19 |
| Effort > reward | 193 | 983 | 2.04 | ^***^ | 1.49-2.80 |  | . |  | . |  | 2.08 | ^***^ | 1.50-2.89 |
| Note. ^+^ *p* < .1, ^*^ *p* < .05, ^**^ *p* < .01, ^***^ *p* <.001. Adjusted models I and II control for the following potential confounders and modifiers: age, educational level, monthly family income, type of the cohort, body mass index, BMI, physical activity, current smoking, alcohol, and noise sensitivity. | | | | | | | | | | | | | |

**Supplementary Table 2** The effect of bedroom window orientation and work-related stress on problems falling asleep, within subsample B, showing univariate and adjusted Odds Ratios (*OR*) with 95 % Confidence Intervals (*CI*).

|  | Problems falling asleep | | Univariate model | | |  | Adjusted model I  (*job strain*) | | |  | Adjusted model II  (*ERI*) | | | |
| --- | --- | --- | --- | --- | --- | --- | --- | --- | --- | --- | --- | --- | --- | --- |
| Variable and level | *n* with  problems | *n* without problems | *OR* | | 95 % CI |  | *OR* | | 95 % CI |  | *OR* | | 95 % CI | |
| *Bedroom window orientation* |  |  |  |  |  |  |  |  |  |  |  |  | |  |
| No street (reference) | 110 | 973 | 1.00 |  |  |  | 1.00 |  |  |  | 1.00 |  | |  |
| Street with low traffic | 39 | 401 | 0.86 |  | 0.59-1.26 |  | 0.78 |  | 0.53-1.16 |  | 0.80 |  | | 0.54-1.19 |
| Street with medium  or high traffic | 18 | 118 | 1.35 |  | 0.79-2.30 |  | 1.07 |  | 0.61-1.86 |  | 1.13 |  | | 0.65-1.98 |
|  |  |  |  |  |  |  |  |  |  |  |  |  | |  |
| *Job strain* |  |  |  |  |  |  |  |  |  |  |  |  | |  |
| Medium/balanced (reference) | 86 | 569 | 1.00 |  |  |  | 1.00 |  |  |  | . |  | |  |
| Low | 46 | 742 | 0.41 | ^***^ | 0.28-0.60 |  | 0.43 | ^***^ | 0.29-0.63 |  | . |  | | . |
| High | 35 | 181 | 1.28 |  | 0.83-1.96 |  | 1.16 |  | 0.74-1.82 |  | . |  | | . |
|  |  |  |  |  |  |  |  |  |  |  |  |  | |  |
| *ERI* |  |  |  |  |  |  |  |  |  |  |  |  | |  |
| Balance (reference) | 31 | 496 | 1.00 |  |  |  | . |  |  |  | 1.00 |  | |  |
| Effort < reward | 22 | 276 | 1.28 |  | 0.72-2.25 |  | . |  | . |  | 1.43 |  | | 0.80-2.54 |
| Effort > reward | 114 | 720 | 2.53 | ^***^ | 1.68-3.83 |  | . |  | . |  | 2.53 | ^***^ | | 1.65-3.87 |
| Note. ^+^ *p* < .1, ^*^ *p* < .05, ^**^ *p* < .01, ^***^ *p* <.001. Adjusted models I and II control for the following potential confounders and modifiers: age, educational level, monthly family income, type of the cohort, body mass index, BMI, physical activity, current smoking, alcohol, and noise sensitivity. | | | | | | | | | | | | | | |

**Supplementary Table 3** Effect of nocturnal road noise exposure and work-related stress on waking up too early and having problems falling asleep again within subsample A, showing univariate and adjusted Odds Ratios (*OR*) with 95 % Confidence Intervals (*CI*).

|  | Waking up and having problems falling asleep | | Univariate model | | |  | Adjusted model I  (*job strain*) | | |  | Adjusted model II  (*ERI*) | | |
| --- | --- | --- | --- | --- | --- | --- | --- | --- | --- | --- | --- | --- | --- |
| Variable and level | *n* with  problems | *n* without problems | OR | | 95 % CI |  | OR | | 95 % CI |  | OR | | 95 % CI |
| *Nocturnal road noise* |  |  |  |  |  |  |  |  |  |  |  |  |  |
| Low (< 45 dB, reference) | 158 | 551 | 1.00 |  |  |  | 1.00 |  |  |  | 1.00 |  |  |
| Medium (45 - 50 dB) | 130 | 533 | 0.85 |  | 0.66-1.10 |  | 0.89 |  | 0.68-1.17 |  | 0.91 |  | 0.69-1.19 |
| High (> 50 dB) | 114 | 605 | 0.66 | ^**^ | 0.50-0.86 |  | 0.71 | ^*^ | 0.54-0.94 |  | 0.72 | ^*^ | 0.55-0.96 |
|  |  |  |  |  |  |  |  |  |  |  |  |  |  |
| *Job strain* |  |  |  |  |  |  |  |  |  |  |  |  |  |
| Medium/balanced (reference) | 198 | 746 | 1.00 |  |  |  | 1.00 |  |  |  | . |  |  |
| Low | 107 | 716 | 0.56 | ^***^ | 0.44-0.73 |  | 0.61 | ^***^ | 0.47-0.79 |  | . |  | . |
| High | 97 | 227 | 1.61 | ^**^ | 1.21-2.14 |  | 1.67 | ^***^ | 1.24-2.25 |  | . |  | . |
|  |  |  |  |  |  |  |  |  |  |  |  |  |  |
| *ERI* |  |  |  |  |  |  |  |  |  |  |  |  |  |
| Balance (reference) | 93 | 545 | 1.00 |  |  |  | . |  |  |  | 1.00 |  |  |
| Effort < reward | 29 | 246 | 0.69 |  | 0.44-1.08 |  | . |  | . |  | 0.79 |  | 0.50-1.24 |
| Effort > reward | 280 | 898 | 1.83 | ^***^ | 1.41-2.36 |  | . |  | . |  | 1.66 | ^***^ | 1.27-2.18 |
| Note. ^+^ *p* < .1, ^*^ *p* < .05, ^**^ *p* < .01, ^***^ *p* <.001. Adjusted models I and II control for the following potential confounders and modifiers: age, educational level, monthly family income, type of the cohort, body mass index, BMI, physical activity, current smoking, alcohol, and noise sensitivity. | | | | | | | | | | | | | |

**Supplementary Table 4** The effect of bedroom window orientation and work-related stress on waking up too early and having problems falling asleep again, within subsample B, showing univariate and adjusted Odds Ratios (*OR*) with 95 % Confidence Intervals (*CI*).

|  | Waking up and having problems falling asleep | | Univariate model | | |  | Adjusted model I  (*job strain*) | | |  | Adjusted model II  (*ERI*) | | | |
| --- | --- | --- | --- | --- | --- | --- | --- | --- | --- | --- | --- | --- | --- | --- |
| Variable and level | *n* with  problems | *n* without problems | *OR* | | 95 % CI |  | *OR* | | 95 % CI |  | *OR* | | 95 % CI | |
| *Bedroom window orientation* |  |  |  |  |  |  |  |  |  |  |  |  | |  |
| No street (reference) | 162 | 921 | 1.00 |  |  |  | 1.00 |  |  |  | 1.00 |  | |  |
| Street with low traffic | 76 | 364 | 1.19 |  | 0.88-1.60 |  | 1.19 |  | 0.87-1.62 |  | 1.25 |  | | 0.92-1.71 |
| Street with medium  or high traffic | 29 | 108 | 1.53 | ^+^ | 0.98-2.38 |  | 1.45 |  | 0.91-2.3 |  | 1.55 | ^+^ | | 0.97-2.46 |
|  |  |  |  |  |  |  |  |  |  |  |  |  | |  |
| *Job strain* |  |  |  |  |  |  |  |  |  |  |  |  | |  |
| Medium/balanced (reference) | 128 | 526 | 1.00 |  |  |  | 1.00 |  |  |  | . |  | |  |
| Low | 88 | 701 | 0.52 | ^***^ | 0.38-0.69 |  | 0.58 | ^***^ | 0.43-0.79 |  | . |  | | . |
| High | 51 | 166 | 1.26 |  | 0.87-1.83 |  | 1.32 |  | 0.89-1.95 |  | . |  | | . |
|  |  |  |  |  |  |  |  |  |  |  |  |  | |  |
| *ERI* |  |  |  |  |  |  |  |  |  |  |  |  | |  |
| Balance (reference) | 66 | 463 | 1.00 |  |  |  | . |  |  |  | 1.00 |  | |  |
| Effort < reward | 28 | 270 | 0.73 | ^***^ | 0.46-1.16 |  | . |  | . |  | 0.80 |  | | 0.50-1.30 |
| Effort > reward | 173 | 660 | 1.84 |  | 1.35-2.50 |  | . |  | . |  | 1.71 | ^**^ | | 1.24-2.36 |
| Note. ^+^ *p* < .1, ^*^ *p* < .05, ^**^ *p* < .01, ^***^ *p* <.001. Adjusted models I and II control for the following potential confounders and modifiers: age, educational level, monthly family income, type of the cohort, body mass index, BMI, physical activity, current smoking, alcohol, and noise sensitivity. | | | | | | | | | | | | | | |
